# A combined modelling approach to predicting injury severity in rear-end collisions

**DOI:** 10.1016/j.mex.2025.103612

**Published:** 2025-09-08

**Authors:** Shufeng Wang, Shixuan Jiang, Zhengli Wang, Lingyi Meng

**Affiliations:** College of Transportation, Shandong University of Science and Technology, Qianwan gang Road, Qingdao 266590, Shandong Province, China

**Keywords:** Rear-end collision, Crash analysis, Severity prediction model, CNN, XGBoost, Combined model

## Abstract

Rear-end collisions constitute the most prevalent category of urban road traffic accidents, resulting in severe traffic congestion, casualties, and substantial economic losses. To mitigate the impact of such accidents effectively, this study proposes a severity prediction model that integrates Convolutional Neural Networks (CNN) and Extreme Gradient Boosting (XGBoost). The model employs the U.S. Department of Transportation's Fatality Analysis Reporting System (FARS) accident dataset, which undergoes preliminary preprocessing. Subsequently, Principal Component Analysis (PCA) is applied to reduce the dimensionality of the influencing factors prior to their input into the combined model for classification. CNN is utilized to extract features, while XGBoost is responsible for classification. Experimental results demonstrate that the combined model achieves a classification accuracy of 96.2 %, with superior AUC and F1 scores compared to traditional models, indicating excellent predictive performance.

• This paper proposes a hybrid CNN-XGBoost algorithm that combines the superior feature extraction capability of CNN with the powerful structured data processing and precise prediction ability of XGBoost, resulting in a significant performance improvement over traditional algorithms.

## Graphical abstract

**Figure:**
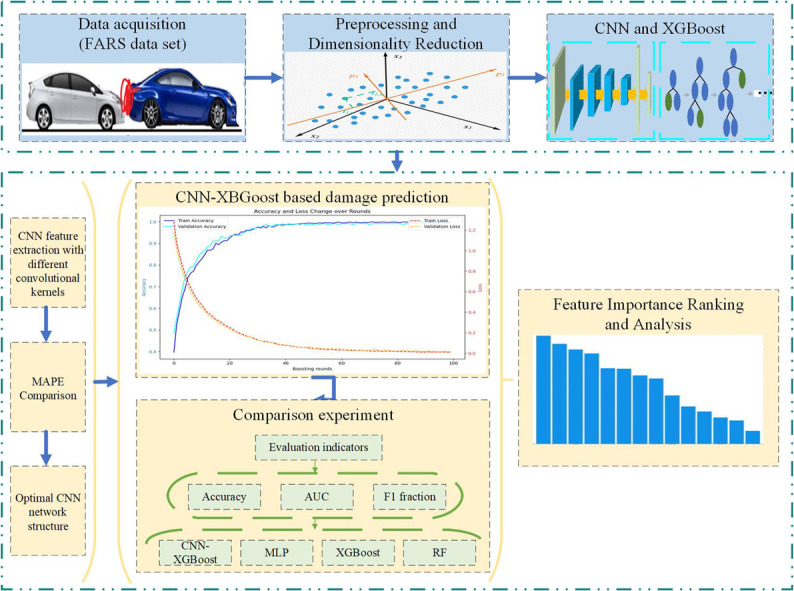

**Specifications table**

**Table:** 

**Subject area**	Computer Science
**More specific subject area**	*Machine Learning*
**Name of your method**	*CNN-XGBoost combined model*
**Name and reference of original method**	*N/A*
**Resource availability**	*Datasets available:*https://www.nhtsa.gov/research-data/fatality-analysis-reporting-system-fars.

## Background

With the rapid development of transportation systems, road traffic accidents have become a critical issue globally. Rear-end collisions, in particular, represent one of the most frequent and severe types of accidents, accounting for approximately 30 % of all incidents, with 89 % attributed to driver errors [[1], [2], [3]]. Existing studies typically employ three types of models to predict accident severity: statistical models, machine learning models, and hybrid models [[4], [5], [6], [7], [8], [9]].

Statistical models such as Logit, Probit, and Logistic Regression (LR) offer high interpretability and have been widely used in severity prediction [10,11]. However, they rely heavily on assumptions of linearity and independence, which limits their ability to capture complex interactions among variables, often leading to poor generalization and biased estimations when assumptions are violated [12]. In contrast, machine learning models—including Random Forest (RF), Support Vector Machines (SVM), and Extreme Gradient Boosting (XGBoost)—demonstrate superior nonlinear modeling capabilities and have shown significantly better predictive performance [[13], [14], [15]]. Nonetheless, these models often operate as “black boxes,” making it difficult to interpret prediction outcomes and identify key contributing factors [16].

Deep learning methods such as Convolutional Neural Networks (CNN) further enhance modeling capabilities, particularly in processing high-dimensional and complex data. Studies have shown that CNNs outperform traditional models when additional contextual data such as time and weather are included [3,17]. However, deep learning models typically require large datasets and suffer from limited interpretability, raising concerns in practical applications.

Hybrid models attempt to combine the strengths of statistical and machine learning approaches to improve accuracy and interpretability [18,19]. For example, a mixed Logit model was integrated with association rule learning to predict pedestrian crash severity, achieving improved explanatory power [20]. Similarly, hybrid models combining neural networks with traditional methods were developed to enhance prediction performance [21,22].

**Fig. 1:**
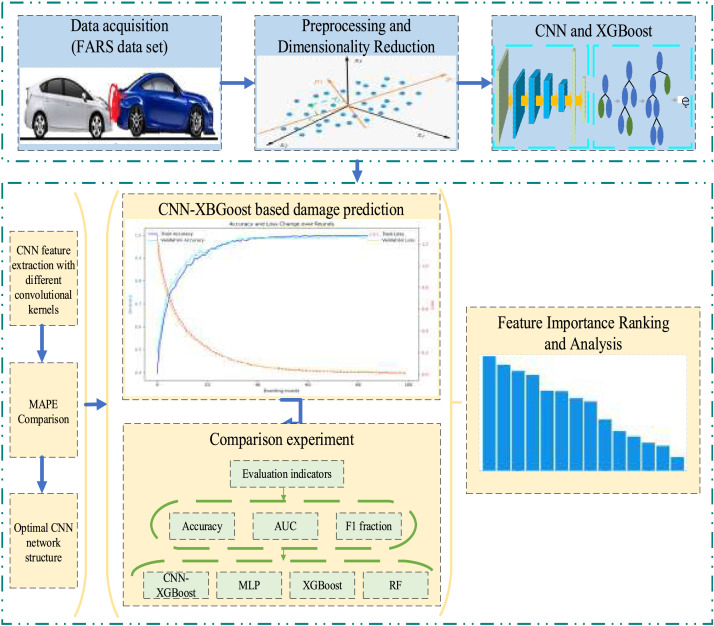
Total framework of the paper.

Given these insights, this study proposes a novel hybrid approach that integrates CNN with XGBoost. CNN is highly effective at learning spatial and hierarchical patterns from complex input features, while XGBoost excels in classification tasks, handling missing data, and reducing overfitting. This combination leverages CNN's feature extraction strengths with XGBoost's robust predictive capabilities. The proposed model is benchmarked against LR, BN, DT, and standalone XGBoost through controlled experiments to support traffic safety decision-making Fig. 1.

## Method details

This study introduces an innovative prediction methodology that amalgamates CNN with XGBoost, thereby capitalizing on their distinctive and complementary attributes. CNN demonstrates remarkable proficiency in processing high-dimensional and intricate datasets, adeptly discerning the interrelationships among diverse factors implicated in rear-end collisions. XGBoost, on the other hand, is renowned for its robustness and superior classification capabilities, effectively managing missing values and accommodating a wide array of feature types while mitigating the risk of overfitting and ensuring precise predictions. Through meticulously designed controlled experiments, the proposed hybrid model is benchmarked against established algorithms such as XGBoost, Logistic Regression (LR), Bayesian Networks (BN), and Decision Trees (DT).

### Data acquisition

The Fatality Analysis Reporting System (FARS) is a comprehensive database maintained by the U.S. National Highway Traffic Safety Administration, which meticulously records detailed information on U.S. traffic accidents, including accident time, location, and casualty information. For the purpose of this study, a total of 39,731 accident records related to rear-end collisions on urban roads were extracted from the FARS database.

### Variable processing

In accordance with the principles of relevance, reliability, and operability, records of accidents caused by drivers' own factors (such as drunk driving and drugged driving) and information unrelated to the severity of accidental injuries (such as license plate number and vehicle manufacturer) were excluded from the dataset. Ultimately, eighteen influencing factors were selected for analysis, with the meanings of each variable detailed in Table 1.

**Table 1:** Description of independent variables.

Name	Description	Typology
AGE	Age of the injured	Continuity
Speed	Relative vehicle speeds at the time of the collision	Continuity
FIRE	Crash fires (0 fires, 1 fire)	Discreteness
AIR_BAG	Airbags (0 not used, 1 used)	Discreteness
REST_USE	Seat belts (0 used, 1 not used)	Discreteness
WKDY	Date (0 working days, 1 rest day)	Discreteness
VALIGN	Lane curvature (0 straight, 1 curved)	Continuity
SPEEDREL	Whether speeding (0 not speeding, 1 speeding)	Discreteness
VPROFILE	Driveway gradient (0 horizontal, 1 sloped, 2 hilly)	Continuity
BDYTYP	Vehicle type (0 regular sedan, 1 mid-size sedan, 2 SUV\truck)	Discreteness
PCRASH	Obstacle avoidance maneuvers in case of collision (0 deceleration, 1 steering, 2 deceleration + steering)	Discreteness
VTRAFWAY	Lanes (0 single lane, 1 two-lane without segregation, 2 two-lane and above)	Discreteness
VTRAFCON	Control equipment (0 no signals, 1 with traffic signals, 2 regulatory warning signs)	Discreteness
GVWR	Vehicle weight (0 for <3001lbs, 1 for 3001–4501, 2 for >4501lbs)	Continuity
WEATHR	Weather (0 sunny, 1 foggy, 2 rainy, 3 snowy)	Discreteness
LGTCON	Light (0 daytime, 1 dawn or dusk, 2 nighttime with light, 3 nighttime without light)	Continuity
VSURCOND	Surface conditions (0 dry, 1 wet, 2 waterlogged, 3 snowlogged, 4 frozen)	Discreteness
HOUR	Time (0 is 6:00–9:59AM, 1 is 10:00–2:59PM, 2 is 3:00–5:59PM, 3 is 6:00–8:59PM, 4 is 9:00–5:59AM)	Continuity

The qualitative classification of the severity of road traffic accidents does not adhere to a uniform international standard. Different countries may establish specific divisions based on their unique characteristics. However, the primary considerations generally include the extent of property damage and the severity of casualties. Consequently, in this study, the dependent variable, accident injury severity, is categorized into four levels, as detailed in Table 2.

**Table 2:** Description of dependent variables.

Classification of severity	Value	Explanation of casualties
undamaged	0	No injuries, property damage only
minor injuries	1	Minor injuries other than fatal and disabling injuries
seriously hurt	2	Non-fatal injuries with varying degrees of disability
dead	3	Death on the spot or within 30

### Normalization

Normalization is an important method of data preprocessing aimed at eliminating scale differences between different features. It achieves this through linear transformation of the original data, mapping the results to the range [−1,1]. This ensures that the impact of each feature on the model is more balanced, preventing certain features with larger value ranges from disproportionately influencing the model. Additionally, normalization helps improve the stability and accuracy of the model while accelerating its convergence. The normalization formula is as follows:

(1) $$x^{*}=\frac{x-x_{min}}{x_{max}-x_{min}}$$

Where $x^{*}$ is the new data obtained by normalization, x is the original data, $x_{\text{min}}$ is the minimum value in the sample data, and $x_{\text{max}}$ is the maximum value of the sample data.

### PCA downscaling

PCA is a widely used data dimensionality reduction algorithm. In data analysis, when there are too many features affecting the indicators, it can lead to overly complex models and the problem of dimensional catastrophe, which affects the validity of the prediction results. For the 18 categories of variables studied, samples from each category were screened to form a total matrix. PCA is performed to find the PCA transformed Y matrix of the X matrix. The covariance matrix of the samples needs to be found, firstly for each vector its eigenmeans are calculated:

(2) $$u=\frac{1}{n}\sum_{i=1}^{n}x_{ij}$$

The covariance matrix S can be obtained from the eigenmeans:

(3) $$S=\frac{1}{n-1}\sum_{i=1}^{n}\left(x_{i}-u\right){\left(x_{i}-u\right)}^{T}$$

The eigenvalues and eigenvectors of the covariance matrix S can be found by using Eq. (4):

(4) $$Sv_{i}=\lambda_{i}v_{i}\;i=1,2,\ldots ,n$$

Where: λ is the eigenvalue of the covariance matrix and also denotes the value of the variance obtained by the ith component, and v is the eigenvector representing the direction of the distribution of the total variance over the ith component.

For each component, the size of its variance as a proportion of the total variance indicates how much information it carries, and from this, the contribution of each component can be derived.

(5) $$P_{i}=\frac{\lambda_{i}}{\sum_{i=1}^{\lambda_{i}}\lambda_{i}}=\frac{\lambda_{i}}{m}$$

**Fig. 2:**
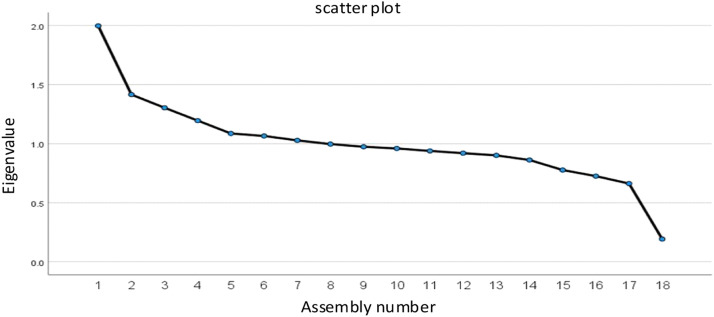
Scatter plot.

Usually, the cumulative contribution rate of the principal components reaches 85 % to fully reflect the information of the original influencing factor variables. The influencing factors of accidental injury severity were analyzed by principal components to obtain the corresponding eigenvalue, contribution rate, and cumulative contribution rate of each principal component, as shown in Table 3. It shows that the cumulative contribution rate of the first 14 principal components is 86.905 %. Therefore, selecting the first 14 principal components to characterize the accident data information contained in the features ensures the mutual independence of each variable in the accident data samples and achieves the effect of dimensionality reduction Fig. 2.

**Table 3:** Interpretation of total variance of principal components.

Ingredient	Eigenvalue			Extract the sum of the squares of the loads		
	Total	Percentage of variance	Cumulative ( %)	Total	Percentage of variance	Cumulative ( %)
$x_{1}$	1.998	11.097	11.097	1.998	11.097	11.097
$x_{2}$	1.416	7.865	18.962	1.416	7.865	18.962
$x_{3}$	1.304	7.245	26.207	1.304	7.245	26.207
$x_{4}$	1.195	6.640	32.847	1.195	6.640	32.847
$x_{5}$	1.086	6.036	38.883	1.086	6.036	38.883
$x_{6}$	1.065	5.918	44.801	1.065	5.918	44.801
$x_{7}$	1.028	5.711	50.511	1.028	5.711	50.511
$x_{8}$	.997	5.537	56.049	.997	5.537	56.049
$x_{9}$	.974	5.411	61.460	.974	5.411	61.460
$x_{10}$	.959	5.330	66.790	.959	5.330	66.790
$x_{11}$	.938	5.213	72.003	.938	5.213	72.003
$x_{12}$	.920	5.109	77.112	.920	5.109	77.112
$x_{13}$	.901	5.006	82.118	.901	5.006	82.118
$x_{14}$	.862	4.787	86.905	.862	4.787	86.905
$x_{15}$	.777	4.317	91.222			
$x_{16}$	.725	4.029	95.251			
$x_{17}$	.662	3.680	98.930			
$x_{18}$	.193	1.070	100.000

Since the principal components cannot visually represent the influencing factors of accident severity, it is necessary to convert the dimensionality reduction results into representative original features to perform subjective analysis and validation of the influencing factors. Based on the eigenvalues and cumulative contributions, the principal component composite scores were calculated, and the explanatory variables SPEED, PCRASH, GVWR, BDYTYP, FIRE, REST_USE, AIR_BAG, HOUR, WKDY, LGTCON, WEATHR, VTRAFWAY, VALIGN, and AGE were determined to be representative of the accident data samples. These 14 variables were utilized for further prediction studies of injury severity. The definitions of the above variables are shown in Table 1.

### CNN

CNN has unique advantages in data processing. One of its main features is local awareness. Through the operation of the convolutional layer, CNN can effectively focus on and extract features from local regions in the data. This approach helps to recognize patterns and associations in the data. Additionally, the parameter-sharing property of CNN enables the network to use the structural information of the data more effectively in the learning process, reducing the number of parameters to be trained and improving the efficiency and generalization ability of the model.

**Fig. 3:**
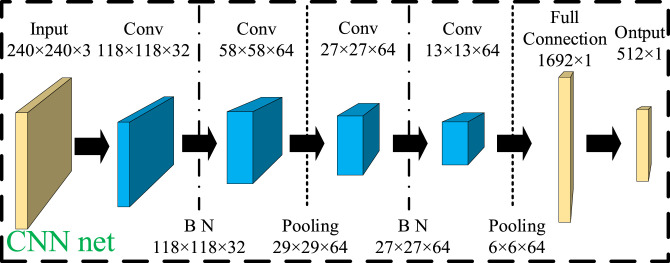
CNN network structure.

The CNN structure is depicted in Fig. 3. The Input layer receives the input, while the Convolution layer (Conv) is responsible for extracting features from the data. Batch Normalization (BN) is utilized to enhance the speed and stability of neural network training. The Pooling layer (Pooling) reduces the dimensionality of the data while retaining key information. The Activation function increases the expressive ability of the model. Lastly, the Full Connection layer (Full Connection) maps the extracted features to the final output. These layers collaborate to construct a deep Learning model that efficiently processes and learns from complex data.

CNN network reduces the high dimensional data by convolution and pooling layer. The height and width of the data after convolution and pooling layer through CNN network changes as in Eq. (6):

(6) N=(W−F+2P)/S+1

where N is the output size, W is the original size of the data input, P is the size of the edge complementary zeros, S is the step size of the convolution kernel move, and F is the size of the convolution kernel.

### Feature extraction

CNN contains a convolutional layer, pooling layer, fully connected layer, and output layer, where neurons in adjacent layers are connected and there is no connection between the same layers. The whole can be divided into three parts: input, feature extraction, and output. The feature extraction stage mainly comprises two parts: convolution and pooling. The entire network can automatically extract features from the input matrix without expert experience.

Convolutional operations leverage the local connectivity property to extract local features of the input matrix. During iterative learning, the shared weights in the convolution kernel are adjusted by gradient descent to maximize the extraction of data features. The convolution is calculated as shown in Eq. (7).

(7) $$X_{ij}=f\left(\sum_{q=1}^{r}\sum_{p=1}^{r}\left(D_{\left(i+p\right)\left(j+q\right)}C_{pq}\right)+b_{c}\right)$$

where $X_{ij}$ is the output feature at position (i,j), $D_{\left(i+p)(j+q\right)}$ denotes the input element, $C_{pq}$ is the convolution kernel weight, $b_{c}$ is the bias term, and f(·) is the activation function applied to the weighted sum.

In this paper, the ReLU activation function (f(x)=max(0,x)) with fast convergence speed is selected, the input matrix D is of dimension n×m, and the sliding calculation with step size 1 is performed using a convolution kernel of r×r, together with the bias variable b to obtain the feature vector of dimension (n−r+1)×(m−r+1).

After the features are extracted from the convolutional layer, the pooling layer is employed for feature aggregation to decrease the computational load and reduce the size of the convolutional features. Pooling is calculated as shown in Eq. (8).

(8) $${\hat{X}}_{ij}=\alpha \times Down\left(X_{ij}\right)+b_{p}$$

Where: Down is the pooling operation, α is the pooling weight and $b_{p}$ is the pooling layer bias weight. The commonly used pooling operations are mean−pooling and max−pooling. denotes that at a pooling scale of l×l, a range of l×l is selected in the convolutional feature matrix for the operation. mean−pooling is replaced by calculating the mean of the range, while max−pooling is replaced by the maximum of the range.

To enhance the effectiveness of the features extracted by CNN, this paper conducts feature extraction using different convolutional kernels and hyperparameters. It selects the appropriate structure and optimal hyperparameters based on the model's fitting ability. Table 4 displays the Mean Absolute Percentage Error (MAPE) of the training set under different convolutional kernels.

**Table 4:** MAPE with different convolutional kernels.

Group	$C_{1}$	$S_{1}$	$C_{2}$	$S_{2}$	MAPE( %)
1	3×3	2×2	3×3	2×2	11.3
2	3×3	2×2	5×5	2×2	14.7
3	5×5	2×2	3×3	2×2	15.1
4	5×5	2×2	5×5	2×2	19.6

As evident from Table 4, the MAPE value is the lowest under combination 1. Therefore, in this paper, group 1 is chosen for convolutional network feature extraction, and the corresponding optimal hyperparameter settings are displayed in Table 5.

**Table 5:** CNN optimal hyperparameters.

Name	Optimizer	Dropout Rate	Learning Rate	Batch Size	Dense_units	Padding
Value	Adam	0.3	0.01	64	256	Valid

### XGBoost

**Fig. 4:**
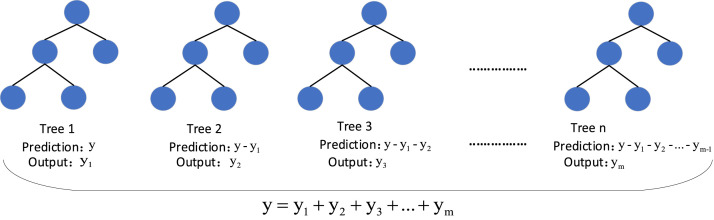
Example of the principle of XGBoost algorithm.

XGBoost is a parallel regression tree model that incorporates the boosting concept, improved based on the gradient boosted decision tree (GBDT) [23]. Compared to the GBDT model, XGBoost overcomes the limitations in computational speed and accuracy with its regularization technique to prevent model overfitting. Unlike traditional GBDT, which performs a first-order Taylor expansion of the computational loss function, XGBoost performs a second-order Taylor expansion to ensure the accuracy of the model. Additionally, each feature is chunked (blocked) and sorted, enabling parallelization of the computation when searching for the optimal splitting point. This greatly accelerates computational speed. Fig. 4 illustrates an example of the principle of the XGBoost algorithm.

The prediction expression is:

(9) $${\hat{y}}_{i}^{\left(t\right)}=\sum_{k=1}^{t}f_{k}\left(x_{i}\right)={\hat{y}}_{i}^{\left(t-1\right)}+f_{t}\left(x_{i}\right)$$

Where: ${\hat{y}}_{i}^{\left(t\right)}$ is the prediction result of sample i after the tth iteration; ${\hat{y}}_{i}^{\left(t-1\right)}$ is the prediction result of the t-1st tree; $f_{t}\left(x_{i}\right)$ is the prediction result of the model for the tth tree.

The objective function of the model consists of a loss function and a regularization term.

(10) $$Obj^{\left(t\right)}=\sum_{i=1}^{n}l\left(y_{i},{\hat{y}}_{i}^{\left(t\right)}\right)+\sum_{j=1}^{t}\Omega \left(f_{j}\right)$$

Where: $\text{Ob}j^{\left(t\right)}$ is the objective function of the model; $\sum_{i=1}^{n}l\left(y_{i},{\hat{y}}_{i}^{\left(t\right)}\right)$ is the corresponding loss function; and $\sum_{j=1}^{t}\Omega \left(f_{j}\right)$ is the regularization term.

**Fig. 5:**
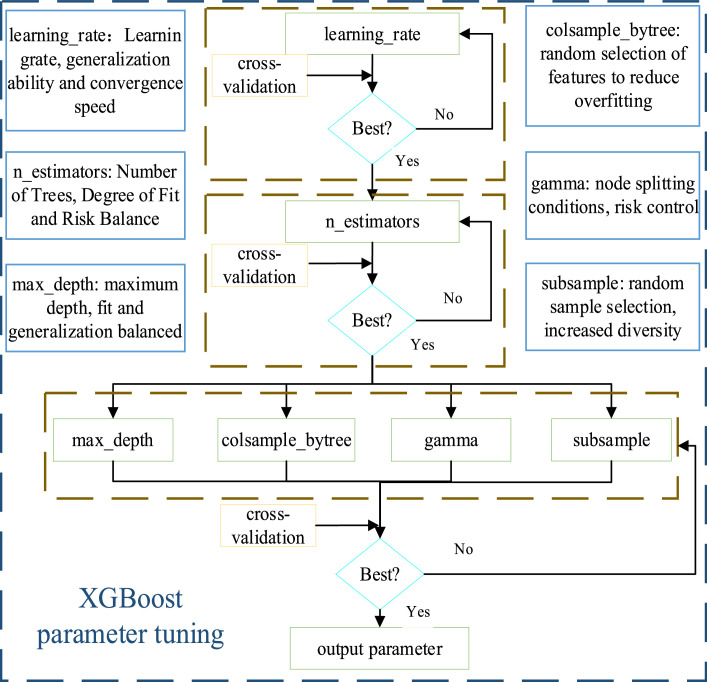
XGBoost hyperparameter optimization flow.

In order to fully utilize the performance of XGBoost, its key parameters need to be adjusted reasonably. These parameters can not only affect the model's fitting ability but also have a significant impact on the accuracy of the prediction results. Therefore, this paper adopts the method of grid search combined with 5-fold cross-validation to tune the key parameters of the XGBoost model to enhance its performance and generalization ability. The parameters optimized in this paper include: n_estimators, learning_rate, max_depth, colsample_bytree, gamma, subsample. The optimization flow is depicted in Fig. 5.

### CNN-XGBoost combination

By combining CNN and XGBoost in a cascade, the complementary strengths of both methods are utilized. This approach leverages CNN's deep learning for automatic feature extraction and XGBoost's ability to combine weak classifiers and rank feature importance. This not only improves prediction performance but also enhances the interpretability of each factor's impact on the results.

**Fig. 6:**
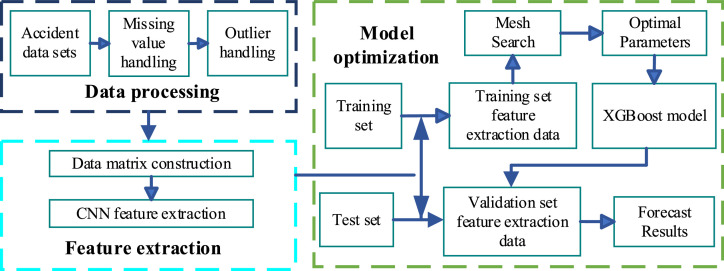
Flow of combinatorial model realization.

The CNN and XGBoost combination model leverages the strengths of both deep learning and GBT algorithms. In this combination, the CNN serves as a feature extractor. A data matrix is constructed and fed into the CNN to extract high-level features. The CNN architecture is then optimized, and the best features are input into the XGBoost model for further prediction and optimization. This approach takes advantage of the CNN's feature extraction capabilities and XGBoost's strength in handling structured data and making accurate predictions. The implementation flow of the combined model is shown in Fig. 6 and Table 6.

**Table 6:** CNN-XGBoost Algorithm Steps.

CNN-XGBoost Algorithm Steps
**Input:** X_train (N_train, 14), y_train (N_train, 1), X_test (N_test, 14), y_test (N_test, 1), Conv_Kernels = [2, 3, 5], XGBoost_Params (search space), K (number of folds for cross-validation), step (grid search step size).
**Output**:Prediction results and performance evaluation metrics.
**Process**:
1. Initialize the optimal CNN model as null and set the minimum MAPE to positive infinity.
2. For each kernel size in **Conv_Kernels**, perform the following steps:
3. Construct a CNN model:
- Design the CNN architecture using the current kernel size.
- Configure convolutional layers, pooling layers, and fully connected layers.
- Compile the CNN model.
4. Perform K-fold cross-validation:
- Split the training data into K folds for training and validation.
- For each fold, train the CNN model and compute MAPE.
- Calculate the average MAPE across all folds.
5. **if** avg_MAPE < min_MAPE:
- Update the optimal kernel size and the minimum MAPE.
6. Construct the optimal CNN model using the selected kernel size, and train it on the entire training set.
7. Apply grid search to optimize the XGBoost hyperparameters defined in **XGBoost_Params**:
8. For each combination of hyperparameters:
- Build an XGBoost model with the current parameter set.
- Evaluate the model and record the performance metrics.
9. Identify the best combination of XGBoost hyperparameters.
10. Feed the optimal features extracted from the CNN into the XGBoost model.
11. Evaluate the performance of the hybrid model on the test set:
- Predict the severity of traffic accidents.
- Assess and record performance metrics such as accuracy, AUC, and F1-score.
12. Output the optimal performance metrics and final prediction results.

### Performance measure

(1) In classification problems, accuracy is one of the most common evaluation criteria, with higher accuracy indicating better model predictions, as shown in Eq. (11).
  (11) $$accuracy=\frac{Number\;of\;Correct\;Predictions}{Total\;Number\;of\;Predictions}$$
(2) Area Under the Curve (AUC) value is one of the metrics used to evaluate the performance of classification models, usually, the AUC value is greater than 0.5, and the closer it is to 1 means that the model performance is better.
(3) The F1 score is an evaluation index that integrates the Precision and Recall of the model, and its value ranges between [0,1], the higher its value, the better the model performs in the classification task of positive and negative categories.
  (12) $$F_{1}=2\;\times \frac{Precision\times Recall}{Precision+Recall}$$

## Method validation

### Model results and interpretation

**Fig. 7:**
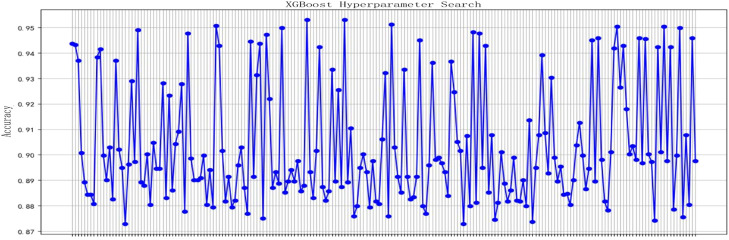
Accuracy with different hyperparameters.

The grid search method is a technique used to adjust the hyperparameters of the model in machine learning. By combining hyperparameter grid search with 5-fold cross-validation, important parameters can be systematically adjusted. This allows for the exploration of various parameter combinations to identify values that optimize the model's performance, thereby enhancing its generalization ability. Consequently, the model can more accurately capture data features, improve prediction performance, and effectively prevent overfitting. Fig. 7 illustrates the accuracy of the training set corresponding to different hyperparameter combinations under the grid search method, the horizontal axis represents the indices of different hyperparameter combinations, while the vertical axis indicates the corresponding training accuracy. Each blue dot represents the training result of a single hyperparameter setting, and the connecting line illustrates the fluctuation trend of accuracy as the hyperparameters change. As observed in the figure, there are significant differences in training accuracy across different hyperparameter combinations, with values ranging approximately from 0.87 to 0.95. This indicates that hyperparameters have a considerable impact on model performance. Some combinations achieve relatively high accuracy (close to 0.95), suggesting that these parameter settings are better suited to the current dataset and enable the model to fit the training data more effectively.

Table 7 displays the values of each parameter for the optimal hyperparameter combination identified through the grid search method after expanding the range of hyperparameter grid.

**Table 7:** XGBoost optimal hyperparameter combinations.

Name	n_estimators	learning_rate	max_depth	colsample_bytree	gamma	subsample	reg_alpha
Value	200	0.1	3	0.4	0.06	0.7	0.2

**Fig. 8:**
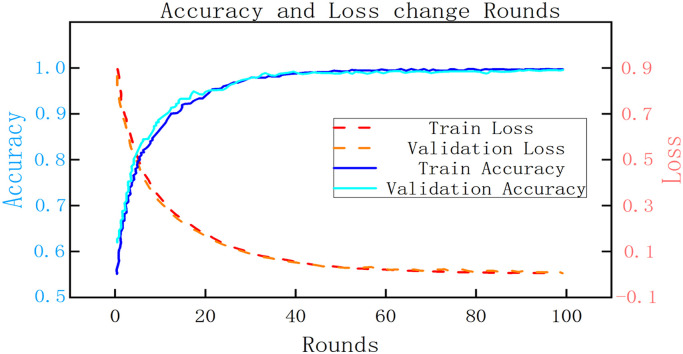
Accuracy and loss variation.

**Fig. 9:**
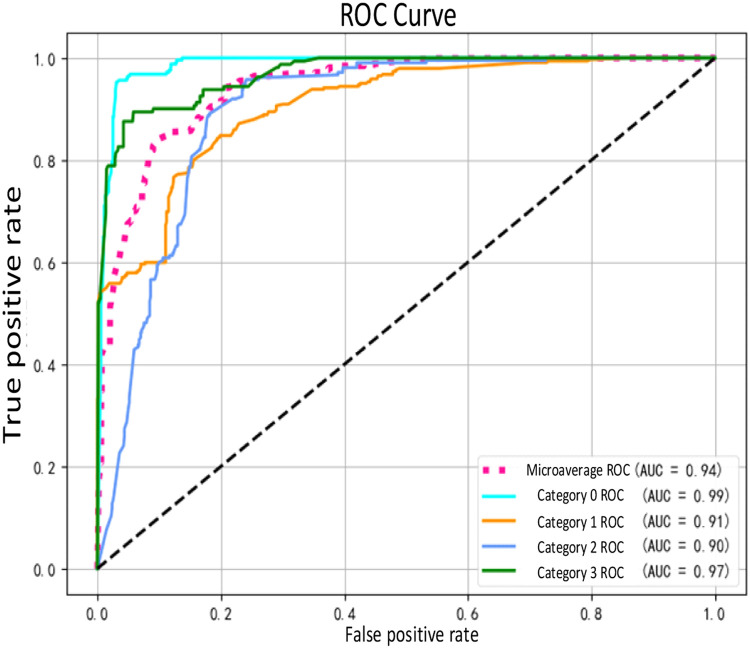
ROC curve.

The optimal model was obtained after completing parameter tuning. At this stage, the model was trained using the training set, and its overall generalization ability and performance were evaluated using the validation set samples. The model's predictive ability for different categories of injuries was assessed using the area under the ROC curves (AUC) values, as depicted in Fig. 8, Fig. 9.

As depicted in Fig. 8, the combined model exhibits a promising prediction performance on the validation set following training with the training set. The average accuracy of the validation set reaches 0.962, indicating excellent generalization ability and rapid convergence. Additionally, the AUC values for the four classifications are 0.99, 0.91, 0.90, and 0.97, respectively. This suggests that the model performs exceptionally well in predicting category 0 (undamaged) and category 3 (dead) incidents, while experiencing a slight decrease in performance for category 1 (minor injuries) and category 2 (serious injuries) predictions due to variations in sample frequency. Upon comprehensive examination of Fig. 8, Fig. 9, the combined model demonstrates an average accuracy of 0.962 and an average AUC value of 0.94, indicating high prediction performance and stability. Thus, the model effectively addresses the rear-end collision prediction task in this study.

### Model comparison

**Fig. 10:**
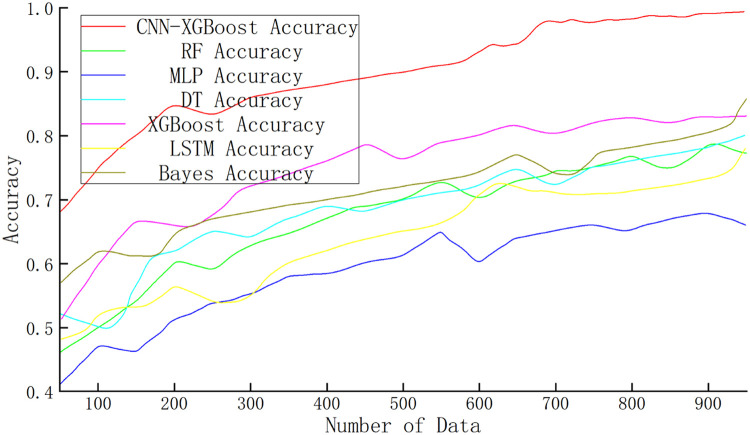
Comparison of the accuracy rates of various models.

To thoroughly evaluate the effectiveness of the combined model, this study conducts a comparative analysis against several widely used algorithms, including XGBoost, RF, and MLP. The performance of each model is assessed across multiple evaluation metrics, such as ROC curves, accuracy, F1-score, and loss variation, to comprehensively measure their effectiveness in rear-end collision risk prediction tasks. Additionally, the AUC is calculated to evaluate the classification performance and generalization ability of each model. Fig. 10 illustrates the comparison results of model accuracy.

As shown in Fig. 10, in the accuracy comparison among the combined model, XGBoost,RF,MLP,DT,LSTM, the combined model demonstrates a significantly higher initial accuracy than the other models. Moreover, as the data volume gradually increases, its accuracy improves at a notably faster rate, indicating superior training efficiency and performance improvement speed. In contrast, the accuracy growth of other models is relatively moderate under the same data conditions, especially for the Multi-Layer Perceptron and Long Short-Term Memory models, which exhibit lower initial accuracy and slower improvement rates.

**Fig. 11:**
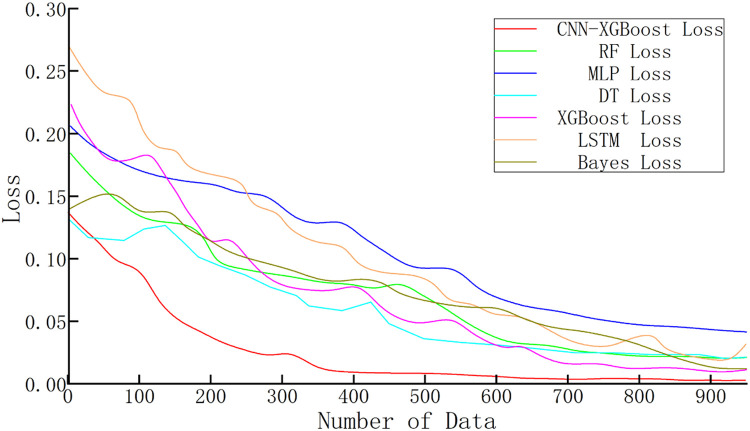
Comparison of the losses of various models.

The variation in loss reflects how well the models fit the validation dataset during training. Lower loss values typically indicate that the model effectively captures patterns in the data, leading to more accurate predictions. The loss variations of each model are illustrated in Fig. 11.

As illustrated in Fig. 11, the convergence speed of the loss function during training varies significantly across the models. The combined model shows a rapid decrease in loss during the early stages and converges to a lower final value, indicating better training efficiency and stronger fitting capability. In contrast, although RF and DT exhibit relatively stable convergence in the later stages, their loss reduction is slower, and the final values remain comparatively high.

Furthermore, the combined model demonstrates a relatively smooth loss decline throughout training. This is due to the CNN's ability to effectively extract key features during the feature extraction phase, enabling XGBoost in the classification stage to converge quickly with fewer training iterations, thus reducing training time and avoiding overfitting. Compared to using XGBoost, MLP, or DT alone, this integrated approach significantly enhances model efficiency in handling complex data, resulting in lower loss values and improved prediction accuracy.

**Fig. 12:**
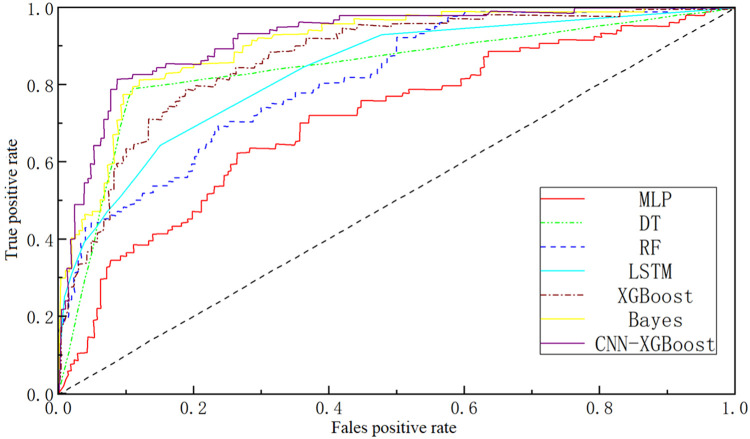
Comparison diagram of the ROC curves of various models.

The micro-averaged ROC curve aggregates TP, FP, TN, and FN across all classes in multi-class classification tasks, providing a comprehensive assessment of overall performance across all categories rather than focusing on a single class. The micro-averaged ROC curves of each model are shown in Fig. 12.

As shown in Fig. 12, the combined model outperforms the other models in terms of the micro-averaged ROC curve, particularly with respect to the AUC. The AUC value of combined model is significantly higher than those of XGBoost, RF, MLP, DT, and LSTM, indicating a stronger ability to distinguish between positive and negative classes—that is, it can more accurately identify the severity level of rear-end collisions.

**Fig. 13:**
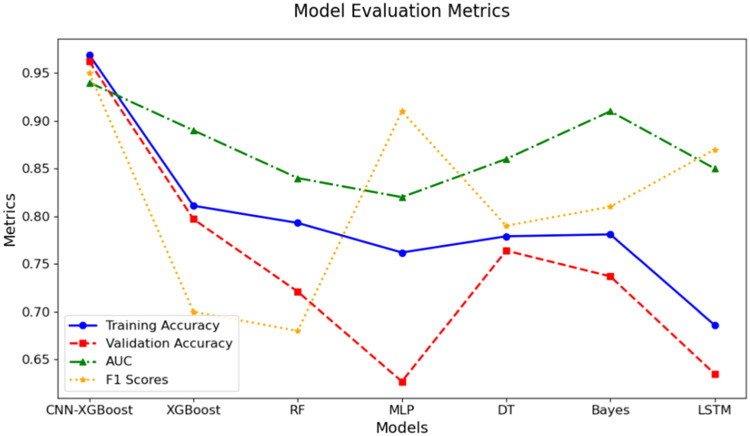
Comprehensive performance comparison.

In addition, the ROC curve of combined model encloses the largest area, suggesting better stability across classification tasks. It consistently maintains high sensitivity and specificity, whereas the other models exhibit fluctuations, indicating relatively poor prediction accuracy in certain categories. This demonstrates that the combined model can handle complex multi-class data more effectively, particularly in high-dimensional tasks such as rear-end collision prediction, by leveraging the feature extraction strength of CNN and the classification power of XGBoost. A comprehensive evaluation of the model's overall performance is presented in Fig. 13 and Table 7.

As observed from the model comparison results in Fig. 13 and Table 8, the training and validation set accuracies, average AUC value, and F1 score of the traffic accident severity prediction model built based on the CNN-XGBoost algorithm are the highest among all models. Compared to the single XGBoost model, integrating CNN with XGBoost yields a significant improvement in overall performance.

**Table 8:** Comparison of performance indicators.

Categorization	Training accuracy	Validation accuracy	AUC	F1 scores
CNN-XGBoost	0.969	0.962	0.94	0.95
XGBoost	0.811	0.797	0.89	0.70
RF	0.793	0.721	0.84	0.68
MLP	0.762	0.627	0.81	0.91
DT	0.779	0.764	0.86	0.79
Bayes	0.781	0.737	0.91	0.81
LSTM	0.686	0.635	0.85	0.87

By comparing the nonlinear machine learning models XGBoost, RF, MLP, DT, Bayes and LSTM algorithms, the average accuracy of the validation set for the combined model is improved by 0.165, 0.241, 0.335,0.198,0.225 and 0.327 respectively. Additionally, the average AUC values are higher by 0.05, 0.1, 0.13,0.08,0.03 and 0.09, indicating that the combined model demonstrates stronger classification abilities and exhibits better robustness when distinguishing between different categories. Moreover, the F1 scores are higher by 0.25, 0.27, 0.04,0.16,0.14 and 0.08 respectively, indicating that the combined model achieves a better balance between accuracy and recall. Compared to a single model, its comprehensive performance is superior when dealing with the same prediction task, suggesting that the constructed combined model possesses excellent adaptability and can demonstrate good prediction performance across various prediction tasks.

## Limitations

In this study, although the rear-end collision severity prediction model based on the CNN-XGBoost algorithm achieved an average accuracy of 0.962 in the experiments, there remains a gap between experimental performance and real-world application. Practical deployment involves a broader range of factors, such as road conditions, weather variations, and driver emotions, many of which were not fully captured by the input variables used in this model. Therefore, future research could improve real-world applicability by tuning additional hyperparameters and incorporating larger, more diverse datasets to better reflect the complexities of actual driving environments.

## Ethics statements

This work did not involve human subjects, animal experiments data, and data collected from social media platforms.

## CRediT authorship contribution statement

**Shufeng Wang:** Conceptualization, Methodology, Data curation, Writing – review & editing. **Shixuan Jiang:** Software, Visualization, Writing – original draft. **Zhengli Wang:** Validation. **Lingyi Meng:** Validation.

## Declaration of competing interest

The authors declare that they have no known competing financial interests or personal relationships that could have appeared to influence the work reported in this paper.

## Data Availability

The data sources used in this study have been provided in the manuscript.
